# Response to Arsenate Treatment in *Schizosaccharomyces pombe* and the Role of Its Arsenate Reductase Activity

**DOI:** 10.1371/journal.pone.0043208

**Published:** 2012-08-17

**Authors:** Alejandro Salgado, Ana López-Serrano Oliver, Ana M. Matia-González, Jael Sotelo, Sonia Zarco-Fernández, Riansares Muñoz-Olivas, Carmen Cámara, Miguel A. Rodríguez-Gabriel

**Affiliations:** 1 Centro de Biología Molecular “Severo Ochoa”, Universidad Autónoma de Madrid-Consejo Superior de Investigaciones Científicas, Madrid, Spain; 2 Departamento de Química Analítica, Facultad de CC. Químicas, Universidad Complutense de Madrid, Madrid, Spain; Texas A&M University, United States of America

## Abstract

Arsenic toxicity has been studied for a long time due to its effects in humans. Although epidemiological studies have demonstrated multiple effects in human physiology, there are many open questions about the cellular targets and the mechanisms of response to arsenic. Using the fission yeast *Schizosaccharomyces pombe* as model system, we have been able to demonstrate a strong activation of the MAPK Spc1/Sty1 in response to arsenate. This activation is dependent on Wis1 activation and Pyp2 phosphatase inactivation. Using arsenic speciation analysis we have also demonstrated the previously unknown capacity of *S. pombe* cells to reduce As (V) to As (III). Genetic analysis of several fission yeast mutants point towards the cell cycle phosphatase Cdc25 as a possible candidate to carry out this arsenate reductase activity. We propose that arsenate reduction and intracellular accumulation of arsenite are the key mechanisms of arsenate tolerance in fission yeast.

## Introduction

Arsenic is a metalloid which is present in the environment both naturally and anthropogenically. As in the case of other metals and metalloids, such as cadmium and chromium, arsenic has been shown to be a health risk at low concentrations. In nature, arsenic is presented in many different oxidation states, being the inorganic ones, arsenite, As (III), and arsenate, As (V), the two main forms. While arsenite is presented mainly in anaerobic and alkaline environments, arsenate is more typical of aerobic and acid environments [1].

According to the World Health Organization (WHO), arsenic poisoning is one of the major health problems in several undeveloped countries, although cases have occurred in countries with a higher level of development [2]. In some areas of countries such as India and Bangladesh, arsenic poisoning is especially worrying and is usually caused by groundwater contamination, reaching levels above 10 µg/L which is the limit established as safe by the United States Environmental Protection Agency (EPA). There is a clear evidence of an association between the intake of arsenic and an increased risk of several types of cancer, miscarriages [3], as well as problems in cognitive development in growth stages [4], [5], [6].

At intracellular level, both arsenate and arsenite work differently. For instance, arsenate can enter the cell via a phosphate transporter due to its structural similarity to phosphate. For the same reason, arsenate can also alter several biochemical reactions such as cellular respiration. On the other hand, several reports have described the capacity of arsenite to damage DNA since arsenite inhibits base- and nucleotide-excision repair mechanisms [7], [8], [9], [10].

Throughout the evolution, several mechanisms of response have been developed by organisms against different stressors. For instance, in the fission yeast *Schizosaccharomyces pombe*, the stress response is mainly directed by MAPKs and more specifically by the Spc1/Sty1 pathway. Spc1 is analogous to mammalian p38, and is activated when different types of stress such as UV radiation, heat shock and hyperosmolarity are present. In addition, it has been described that p38-like pathways are activated in response to arsenic stress in both *S. pombe* and *Saccharomyces cerevisiae* [11], [12].

Regarding arsenate, several reports have shown that MAPK pathway is not the only mechanism of response used by eukaryotic organisms against arsenate. Some organisms can reduce arsenate to arsenite through the activity of arsenate reductases. Arsenite resulting from this reduction is removed from the cell through specific transporters (*Escherichia coli* Arsb, *S. cerevisiae* Acr3p, etc). This reducing capacity has been described in unicellular organisms, such as *Leishmania major* and *S. cerevisiae*, and pluricellular organisms, such as the fern *Pteris vittata* and human [13], [14], [15], [16]. In the latter, arsenic reduction is carried out by the cell cycle phosphatase Cdc25, which also regulates G2/M transition by activating dephosphorylation of CDKs (cyclin dependent kinases) [17].

Studies about the mechanisms of stress response, signal transduction and cell cycle regulation using model organisms such as the yeasts *S. pombe* and *S. cerevisiae* have provided an important framework for investigating analogous mechanisms in higher eukaryotes.

In this report, we will attempt to unravel the intracellular mechanisms established in *S. pombe* in response to arsenic, more specifically to its pentavalent form. The conclusions drawn from this paper, taken in conjunction with previous works, could be useful to achieve a deeper understanding of the mechanisms of arsenic toxicity and detoxification in higher eukaryotes.

## Materials and Methods

### Strains and Media

All strains of *Schizosaccharomyces pombe* used in this study are listed in table 1. All different strains were cultivated in yeast extract medium (YES), at a temperature of 30°C with shaking. Deionized water was used to prepare the media. Media was sterilized in autoclave at 1 atm/121°C for 15 minutes. A spectrophotometer Spectronic 20D (Milton and Roy Company, France) was used to determine the number of cells of each culture (1 OD is about 10^7^ cells/mL).

**Table 1:** Genotypes of *Schizosaccharomyces pombe* strains used in this work.

Strain Name	Genotype	Source
PR109	*h- leu1-32 ura4-D18*	Paul Russell’s laboratory
KS1366	*h+ leu1-32 ura4-D18 spc1::ura4*	Paul Russell’s laboratory
JM544	*h- leu1-32 ura4-D18 wis1::ura4*	Paul Russell’s laboratory
KS2136	*h- leu1-32 ura4-D18 wis4::ura4*	Paul Russell’s laboratory
KS2185	*h- leu1-32 ura4-D18 his7-366 win1-1wik1::his7*	Paul Russell’s laboratory
PR1337	*h- mcs4-13*	Paul Russell’s laboratory
KS1376	*h- spc1:HA6His*	Paul Russell’s laboratory
PS2759	*h- leu1-32 ura4-D18 spc1:HA6His (ura4) wis1::ura4*	Paul Russell’s laboratory
KS1891	*h- leu1-32 ura4-D18 spc1:HA6His (ura4) wis1::myc*	Paul Russell’s laboratory
KS2086	*h- leu1-32 ura4-D18 spc1:HA6His (ura4) wis1-AA::myc*	Paul Russell’s laboratory
KS2149	*h+ leu1-32 ura4-D18 his7-366 spc1:HA6His (ura4) win1-1*	Paul Russell’s laboratory
KS2138	*h- leu1-32 ura4-D18 spc1:HA6His (ura4) wis4::ura4*	Paul Russell’s laboratory
KS2189	*h- leu1-32 ura4-D18 his7-366 spc1:HA6His (ura4) win1-1 wik1::ura4*	Paul Russell’s laboratory
*2209*	*h- leu1-32 ura4-D18 his7-366 spc1:HA6His (ura4) pyp1::leu2 win1-1 wik1::his7*	Paul Russell’s laboratory
MR218	*h- leu1-32 ura4-D18 his7-366 spc1:HA6His (ura4) pyp2::ura4 win1-1 wik1::his7*	Laboratory collection
MR15	*h- ura4-D18 cdc25∶12 myc*	Laboratory collection
GL125	*h- leu1-32 ura4-D18 cdc2-3w*	Paul Russell’s laboratory
MR661	*h-leu1-32 ura4-D18 cdc2-3w cdc25::ura4*	Laboratory collection

### Viability Assays

For plate survival assays, different concentrations of both arsenate and arsenite (25 µM to 100 µM) were added depending on the experiment. Once the culture reached 0.3 OD, serial dilutions of yeast cultures were spotted in plates. Plates were incubated at 30°C for 48–72 hours.

### Stress Treatment of Cells

Cells were cultivated up to 0.3–0.5 OD as explained before and arsenate was added at a final concentration of 100 µM. For immunoblotting analysis and mRNA extraction and quantification, cells were harvested by either filtration or centrifugation, respectively, and immediately stored at −80°C.

### Immunoblotting

To purify the Spc1:HA6His protein we followed previously described protocol [18]. Purified Spc1:HA6His protein was loaded in SDS-PAGE and phosphorylation detected by immunoblotting. Phosphorylation was detected using anti-phospho p38 MAPK antibody (Cell Signaling Technology, USA) and the amount of Spc1:HA6His loaded was measured with an anti-HA antibody (Amersham, USA). Immunoreactive bands were revealed with horseradish peroxidase-conjugated secondary antibodies (Amersham, USA). Cdc25:myc was detected using anti-myc epitope antibodies (Cell Signaling Technollogy, USA) and actin with anti-actin antibodies (MP Biomedicals, USA).

### mRNA Extraction and Quantification

Cells were harvested by centrifugation at OD = 0.5. Both mRNA extraction and purification were performed as previously described [19]. To quantify the amount of Cdc25 mRNA, total RNA was used as template for reverse transcription and preparation of total cDNA (Reverse Transcription System, Promega Corporation, USA). Finally, the *S. pombe cdc25* gene transcription level was determined by a quantitative PCR (qPCR) using that cDNA as template.

### Arsenic Speciation Studies

Arsenic speciation studies were performed as described in [20]. An ultrasonic homogenizer, model SONOPLUS HD 2200 (Bandelin, Germany), equipped with a converter UW 2200, SH 213 G horn as amplifier and sonotrode MS 73 (3 mm titanium microtip) was used for cell extracts treatment. A centrifuge model 5804 Eppendorf (Hamburg, Germany) was used for phase separation after the extraction step.

A Perkin-Elmer 4100 ZL atomic absorption spectrometer with a longitudinal Zeeman background correction, equipped with a transversely heated graphite tube atomizer (THGA) with L’vov platforms was used for arsenic quantification. A Perkin Elmer arsenic electrodeless discharge lamp (EDL) with wavelength 197.3 nm and instrument slit width 0.7 nm was used. A Perkin Elmer EDL System was used to stabilize the lamp current between 349–351 mA. As alternative analytical technique for the determination of As, an ICP-MS HP-7700 Plus (Agilent Technologies, Analytical System, Tokyo, Japan) was used. It was equipped with a Babington nebulizer, Fassel torch and double pass Scott-type spray chamber cooled by a Peltier system. Single ion monitoring at m/z 75 was used for data collection.

The chromatographic system employed for As speciation consisted of a model PU-2080 Plus Pump, (JASCO Corporation, Tokyo, Japan) and PRP-X100 analytical and guard anion-exchange column (Hamilton, Reno, NV, USA). The column effluent was directly introduced into the nebulizer of the ICP/MS previously described via a PTFE capillary tube (0.5 mm i.d.). The samples were injected through a six port-valve (Rheodyne 9125, USA).

### Reagents and Standards Employed for As Analysis

High-purity deionized water (Milli-Q Element system, Millipore, USA) was used for sample and standard solutions preparation. Ten milligrams per liter stock solutions, expressed as metal, of MMA and DMA, were prepared in 4% HNO_3_ by dissolving adequate amounts of CH_3_AsO_3_Na_2_ (MMA) and (CH_3_)_2_AsO_2_Na·3H2O (DMA), both 98% purity from Merck (Darmstadt, Germany). Ten milligrams per liter stock solutions of As (V) and As (III) were prepared fromAs_2_O_5_·2H_2_O (98.5%) from Merck (Darmstadt, Germany) and As_2_O_3_ (99.5%) from J.T. Baker (Deventer, Holland), respectively. All these solutions were kept at 4°C and stored in high density polyethylene (HDPE) bottles until use. Working solutions were prepared daily. The Pd(NO_3_)_2_ matrix modifier solution employed for GFAAS analysis was made from a dilution of 10 g/L Pd solution (Merck, Germany) with water to 2 g/L. HPLC-grade methanol from SDS (Barcelona, Spain) and (NH_4_)H_2_PO_4_ from Merck (Darmstadt,Germany) were the reagents employed as mobile phase of the chromatographic system.

### Samples

The samples prepared were kept frozen (−80°C) until analysis. Total arsenic and arsenic species were determined in two types of samples: yeast *Schizosaccharomyces pombe* extract and yeast extract (YES).

### Analytical Procedures

Total arsenic was determined in *YES* by ZGF AAS by diluting one hundred times and adding five percent of nitric acid to eliminate matrix effects as well as Pd (NO_3_)_2_. It was necessary to modify the thermal furnace program respect to recommended conditions by the manufacturer. The furnace program finally employed is showed in Table 2. A YES volume of 20 µL was injected together with 3 µL of 2 g/L Pd(NO_3_)_2_.

**Table 2:** Graphite furnace programme.

Step	T (°C)	Ramp (s)	Hold (s)	Flow (mL/min)
1	90	5	10	250
2	110	3	20	250
3	300	20	10	250
4	1100	30	20	250
5	1200	1	2	0
6	2100	0	4	0
7	2300	1	4	250

**Figure 1:**
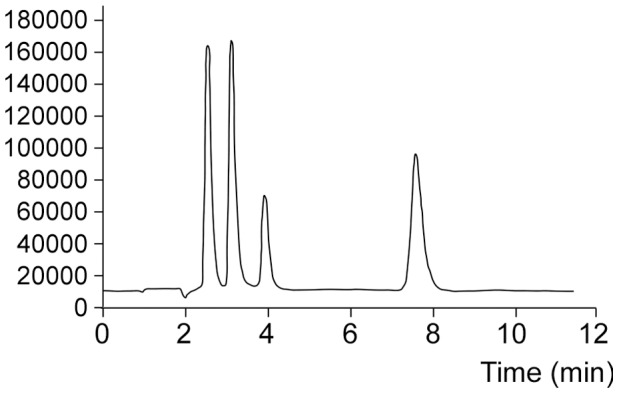
Typical Chromatogram obtained for a standard solution of As species at 2.5 µg L^−1^ using the experimental parameters summarized in Table 3. Peak 1: As (III); Peak 2: DMA; Peak 3: MMA; Peak 4: As (V).

Arsenic speciation was carried out in yeast *Schizosaccharomyces pombe* extracts by LC-ICP/MS. The yeast extracts were diluted two hundred times with deionizer water and introduced into a vial Teflon. The ultrasonic probe was then introduced into the solution and sonication was applied during 30 seconds at 30% amplitude. The extracts were centrifuged at 5000 rpm for 10 minutes and the supernatant was passed through a 0.22 µm nylon syringe filter before analysis. The chromatographic conditions were previously optimized (Sanz et al., 2005). Briefly, a polymeric anion-exchange column, PRP-X100 and mobile phase of 10 mM HPO_4_^−2^/H_2_PO_4_ at pH 8.5 plus 2% of methanol was added to the 10 mM phosphate mobile. The flow rate was 1 mL/min. Under these conditions appropriate separation of the four targeted species (As (III), MMA, DMA and As (V)) in 9 min can be obtained as shown in a typical chromatogram like figure 1. The instrumental parameters for total As determination and speciation analysis have been summarized in table 3.

**Table 3:** Instrumental parameters for As determination by LC/ICP/MS.

ICP MS	
RF power	1550 W
Ar flow rate	Plasma gas: 15 L min-1
	Nebulizer: 1 L min-1
Isotope monitored	75 As
Integration time	0.1 s (spectrum) per point
Points per peak	3
**HPLC**	
Column	PRP-X100 anion Exchange
	Dimensions: 250 mm×4.1 mm, particle size 10 µm
Guard column	PRP-X100 anion exchange
	Dimensions: 4.6 mm
Mobile phase	10 mM HPO_4_^−2^/H_2_PO_4_^−^; 2% (v/v) MeOH; pH 8.5
	100 µL
Flow rate	1.5 mL min-1
Mode	Isocratic

## Results

### Spc1 MAPK Pathway Components are Required for the Response to Arsenate

We have previously described that trivalent arsenic is able to activate the MAPK Spc1 in *Schizosaccharomyces pombe* and cells deficient in this MAPK are sensitive to As (III) [11]. Arsenate, As (V) is the most abundant form of arsenic in many sources of drinking water and is thought to be responsible for many of the chronic effect of arsenic. We decided to study arsenate behavior and compare it with arsenite effects on cellular physiology using a simple eukaryote as *Schizosaccharomyces pombe* as model organism.

**Figure 2:**
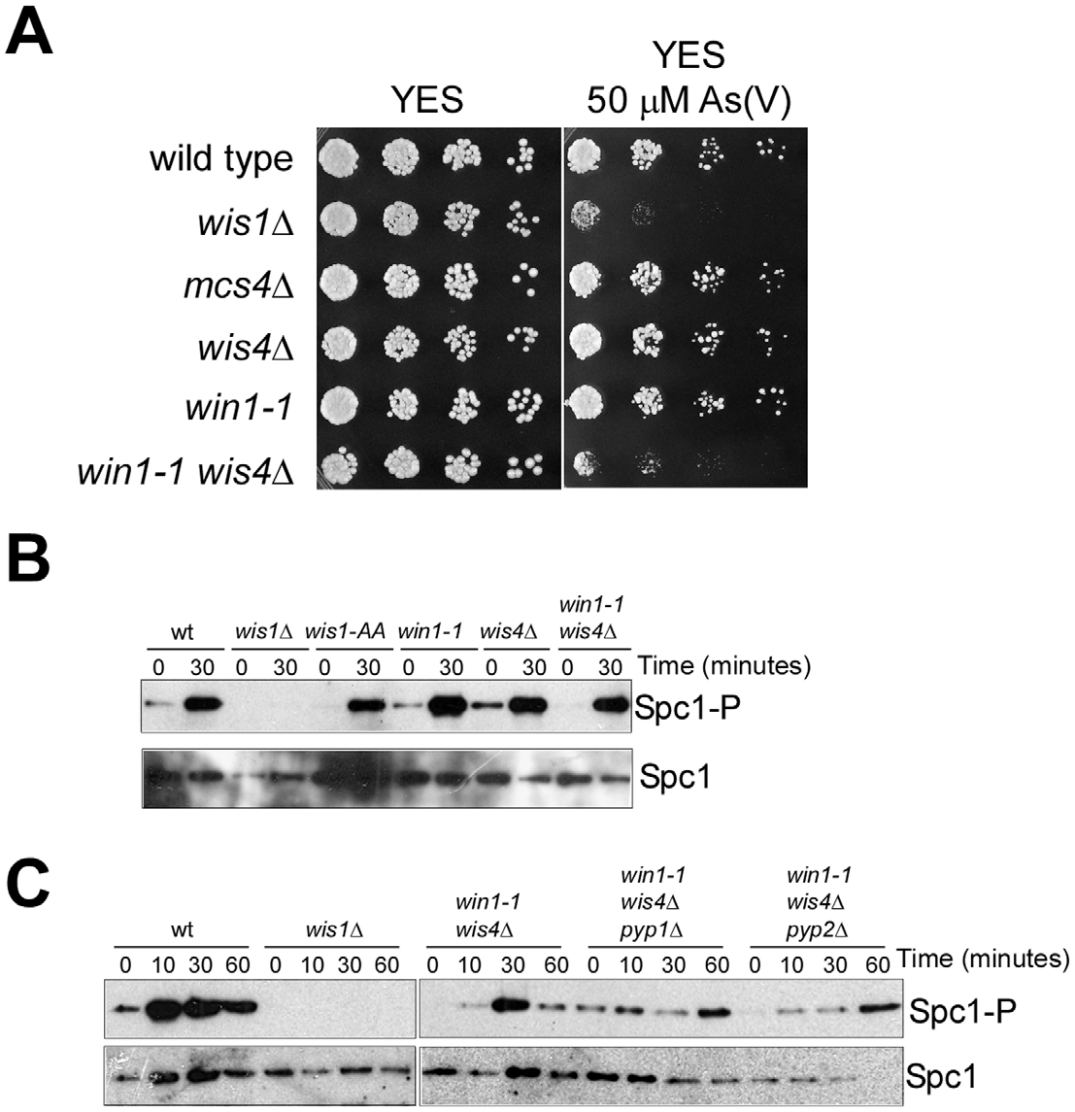
Spc1 MAPK pathway and the response to arsenate. A. Serial dilutions of wild type, *wis1*Δ, *mcs4*Δ, *wis4*Δ, *win1-1* and *wis4*Δ *win1-1* strains were plated in rich media (YES) or rich media containing 50 µM sodium arsenate. Pictures were taken after incubation at 30°C for 48 hours. B. Western blotting of purified Spc1 extracts from wild type, *wis1*Δ, *wis1-AA*, *win1-1, wis4*Δ, and *win1-1 wis4*Δ treated with 100 µM sodium arsenate for 0 to 30 minutes. Antibodies against phosphorylated p38 were used. As a control, antibodies against HA epitope were used. C. Western blotting of purified Spc1 extracts from wild type, *wis1*Δ, *win1-1 wis4*Δ, *win1-1 wis4*Δ *pyp1*Δ and *win1-1 wis4*Δ *pyp2*Δ treated with 100 µM sodium arsenate for 0 to 30 minutes. Antibodies against phosphorylated p38 were used. As a control, antibodies against HA epitope were used.

First, we monitored the sensitivity to arsenate of different fission yeast strains deficient in one or more genes participating in the activation of the MAPK Spc1 (Figure 2A). We compared the viability under chronic exposure to arsenate of those strains using serial dilutions in plates containing rich media.

Using this experimental approach we observed that cells deficient in the MAPKK Wis1 and a double mutant lacking MAPKKKs Wis4 and Win1, were very sensitive to arsenate. However, mutants deficient in Mcs4 or in each one of the MAPKKKs did not show any increased sensitivity to arsenate.

These results indicate that the activation of the MAPK Spc1 is essential for the efficient response to arsenate and the activations requires full function of the MAPKK.

### Activation of Spc1 is Wis1 Dependent but can be Mediated through a MAPKKK Independent Mechanism

We had previously described that arsenite activation of Spc1 was mediated through a mechanism that depends on Wis1 activation, but also on a Wis1-activation independent mechanism [11]. In order to monitor the presence of a similar mechanism after arsenate treatment, we took advantage of different fission yeast mutant strains available. Under arsenate treatment, Spc1 is strongly activated trough a mechanism that requires Wis1 (Figure 2B). However, this activation was still present when the treatment was performed in a mutant strain where both activating phosphorylation sites from Wis1 where changed to the non-phosphorylable aminoacid alanine (Figure 2B). Similar situation was observed in mutants lacking either MAPKKK or both. This result indicates that Spc1 activation under arsenate treatment depends (like in the case of arsenite), on the presence and activation of Wis1, but also on a Wis1-activation independent mechanism.

To further advance in our knowledge of the mechanism of Spc1 activation under arsenate treatment, we also monitored the activation of Spc1 in mutants lacking Wis4 and Win1 activities and each of the phosphatases Pyp1 and Pyp2 (Figure 2C).

We reasoned that if the activation of Spc1 was independent of Wis1 phosphorylation, it may be dependent on Pyp1 or Pyp2 inhibition. As seen in Figure 2C, in mutants lacking Wis4, Win1 and Pyp2 activities, Spc1 activation still occurs in the presence of arsenate, indicating that its activation depends on other mechanism. However, cells lacking Pyp1 activity in a *wis4*Δ *win1-1* genetic background showed a decrease capacity to phosphorylate Spc1 upon arsenate treatment.

This result is consistent with a mechanism where Spc1 regulation is achieved both, through activation of Wis1 and inhibition of Pyp1.

### Fission Yeast Displays Arsenate Reductase Activity

The results described above using arsenate as a stress source, resemble those previously obtained with arsenite. One possible explanation for these similar responses could be that arsenate is transformed into arsenite through a biochemical transformation performed by the fission yeast *Schizosaccharomyces pombe*. However, such arsenate reductase activity has not been described in fission yeast yet.

**Figure 3:**
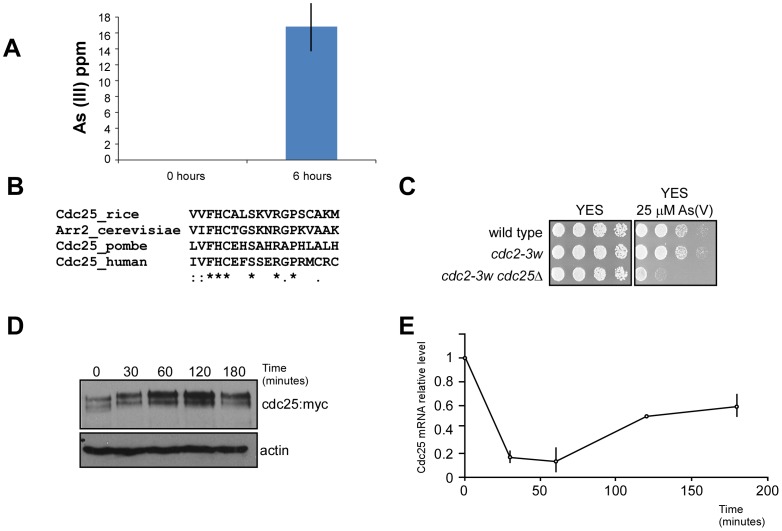
Cdc25 is essential for the response to arsenate. A. Arsenate to arsenite conversion in fission yeast. Cell extracts from cells treated with 100 µM sodium arsenate were analyzed for the presence of As (III) at different time points. Graph represents parts per million (ppm) As (III). B. Protein alignment of a fragment of *S. pombe* Cdc25, rice Cdc25 and *S. cerevisiae* Acr2 and human Cdc25. Asterisks indicate full conservation. C. Serial dilutions of wild type,*cdc2-3w* and *cdc2-3w cdc25*Δ strains were plated in rich media (YES) or rich media containing 25 µM sodium arsenate. Pictures were taken after incubation at 30°C for 48 hours. D. Western blotting of whole cell extracts from Cdc25:myc strains treated with 100 µM sodium arsenate for 0 to 180 minutes. Anti-myc antibodies were used to detect Cdc25:myc and anti-actin as a control. E. Total RNA from the experiment presented in (D) was purified and the total amount of Cdc25 mRNA quantified by qPCR. Actin mRNA was used as an internal control.

We expected that if such arsenate reductase activity existed in fission yeast, intracellular arsenite should appear in the course of an experimental treatment. We obtained whole cell extracts of fission yeast cells treated with As (V) and determined the intracellular concentrations of As (V) and As (III) at different time points. As seen in Figure 3A, intracellular As (III) concentrations increases with time, indicating that the arsenate added to the media has been transformed into arsenite by a cellular activity.

### Cdc25 is Required for Arsenate Response in Fission Yeast

We have determined that fission yeast presents arsenate reductase activity *in vivo*. Our next question was what protein or proteins were carrying out such activity.

One of our approaches was to look for *S. pombe* genes similar to known arsenate reductases in other organisms. We found that arsenate reductases and Cdc25 proteins share similarities in their catalytic domain. We compared *S. pombe* Cdc25 sequence with rice Cdc25 and arsenate reductase Acr2 from *Saccharomyces cerevisiae* and human arsenate reductase Cdc25.

As observed in Figure 3B, the similarity between the 4 proteins in their catalytic domains is very high with a strong conservation in several key aminoacids.

If Cdc25 is an arsenate reductase we would expect that cells deficient in Cdc25 would be sensitive to arsenate. However, Cdc25 is an essential gene that cannot be eliminated in a haploid wild type genetic background because is indispensable for the advance of cell cycle through the dephosphorylation and activation of the CDK, Cdc2. However, it has been described that cells carrying a hyperactive allele of Cdc2, the *cdc2-3w* allele, were able to survive in the absence of Cdc25. We therefore monitored the sensitivity of *cdc2-3w cdc25Δ* strain to arsenate treatment (Figure 3C).

As observed in Figure 3C, cells deficient in Cdc25 were more sensitive to arsenate than wild type or *cdc2-3w* strains. Interestingly the abundance and mobility of Cdc25 protein was altered after arsenate treatment (Figure 3D), and the mRNA encoding Cdc25 also suffers fluctuations after arsenate treatment (Figure 3E), indicating that Cdc25 expression may be regulated by arsenate.

### Arsenate Reductase Activity Requires Wild Type Activity of Spc1, Cdc2 and Cdc25

The results described before indicated that Cdc25 has a role in the response to arsenate, perhaps through its arsenate reductase activity. In order to test this hypothesis, we determined the arsenic species As (III) and As (V) in cellular extracts and growth media obtained from wild type, *spc1*Δ, *cdc2-3w* and *cdc2-3w cdc25*Δ strains after arsenate treatment.

**Figure 4:**
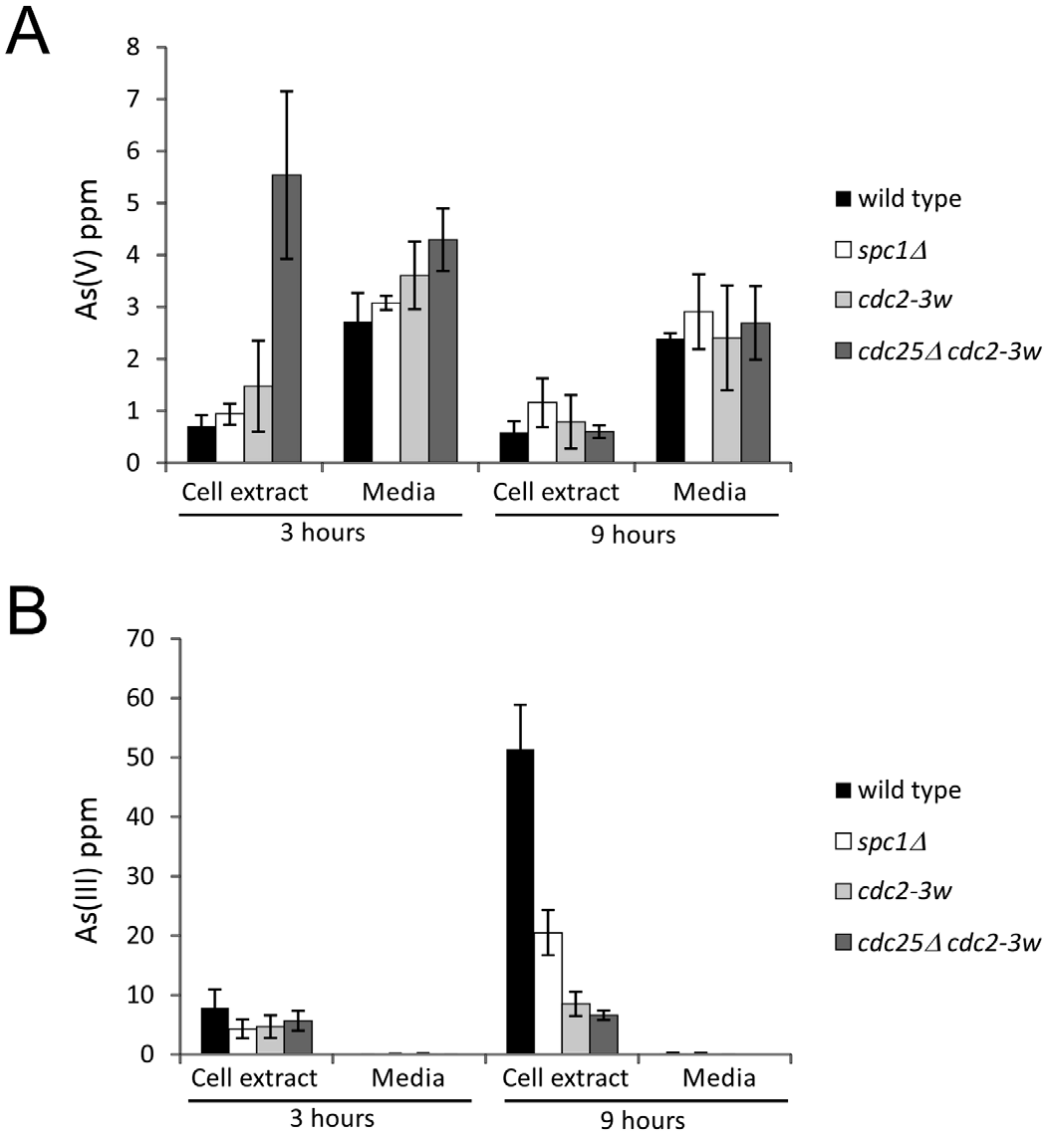
Arsenic speciation in different fission yeast mutants. Total cell extracts from 5×10^7^ cells and growth media from wild type, *spc1*Δ, *cdc2-3w* and *cdc25*Δ *cdc2-3w* strains were obtained after treatment for 3 or 9 hours with 100 µM sodium arsenate. Graph shows the amount of As (V) (A) or As (III) (B) present in the extracts or growth media.

In Figure 4A, As (V) appears to accumulate in cell extracts from wild type and mutant strains treated, like *spc1*Δ, *cdc2-3w* and *cdc2-3w cdc25*Δ strains, after 3 or 9 hours. This accumulation is specially high in mutants lacking Cdc25, that is consistent with a role of this phosphatase in As (V) removal from fission yeast cytoplasm. After 9 hours, all strains have similar As (V) levels in their cytoplasms. During this period (3–9 hours after treatment), the growth media did show a slight decrease in the total concentration of As (V).

In Figure 4B we show the result of quantifying the amount of As (III) in the same experiment. After 3 hours treatment with As (V), a noticeable amount of As (III) appeared in the interior of fission yeast cells. This As (III) resulted from the cellular reduction of As (V) into As (III). The accumulation of As (III) was significantly higher in wild type cells than in the other mutants assayed (Figure 4B, 9 hours). Interstingly, the amount of As (III) present in the cellular exterior (growth media) was detectable, but very low.

Together with the accumulation of As (III), these results indicate that Cdc25 might be required for arsenate reductase activity, and that this activity could be affected by the presence of Cdc2 in the cell.

## Discussion

In this report, several mechanisms by which *S. pombe* is able to respond to arsenate have been analyzed. The overall conclusion from these studies is that the response of fission yeast to arsenate and arsenite is different to the response to other types of stress like, for example, high osmolarity. Besides this, we have described for the first time that *S. pombe* has an arsenate reductase activity. We discuss the possible role of Cdc25 phosphatase as the leading candidate to perform this activity in *S. pombe* and its possible functional interaction with Cdc2 kinase.

### Activation of the Spc1/Sty1 Stress Response Pathway by Arsenate

Viability under arsenate treatment assays have shown that a correct Spc1 MAPKs pathway is essential for cell survival against this type of stress. In contrast to arsenite, *S. pombe* has a much higher sensitivity to arsenate, reaching growth inhibition at micromolar concentrations (This work and [11]).

As observed in Western blotting experiments, it is quite possible that Spc1 could be activated by alternative mechanisms to the MAPK pathway, mainly at level of the MAPKK Wis1. Interestingly, Pyp1 phosphatase appears to be directly involved in this process. The lack of Pyp1 along with a defective MAPK Spc1 pathway does prevent further activation of Spc1 when arsenate is present. In strains with a functional Pyp1, but deficient in Pyp2, activation of Spc1 seems to occur. Therefore, Pyp1 is a good candidate to be inhibited by arsenate *in vivo*.

Comparisons with previous reports studying the role of MAPK pathways in response to arsenic in *S. cerevisiae*, showed similar results to those obtained in our experiments, where MAPK Hog1 is activated in response to arsenite and Slt2 does so in response to arsenate [12], [21]. These results show that this type of arsenic stress response not only appears in *S. pombe*, but has been conserved throughout the evolution, although the mechanisms may be slightly different, at least at the level of MAPK specificity.

### Arsenate Reductase Activity of the Cell Cycle Phosphatase Cdc25

Arsenite found in the arsenic speciation experiments, raises the possible existence of alternative response mechanism to the Spc1 pathway, by which *S. pombe* is able to respond to the stress by arsenate. This mechanism could be the reduction of arsenate into arsenite, ability that has already been described in other organisms [13], [14], [15]. More recently, experiments focused on the human cell cycle phosphatase Cdc25 have also described this reducing capacity for this protein [16].

As observed in our results, arsenate reduction occurs in *S. pombe.* This arsenate reduction activity is affected by the presence of Spc1, Cdc2 and Cdc25.

Like *spc1*Δ strain, *cdc2-3w*, which presents hyperactivated Cdc2, shows a diminished capacity to reduce arsenate into arsenite in the cell. Because of this, it could be assumed an inhibitory role to Cdc2 on arsenate recution activity. Interestingly, Cdc25 activates Cdc2 by removing an inhibitory phosphate previously placed by the kinase Wee1. Given this result, it could be assumed that in the double mutant *cdc2-3w cdc25*Δ the kinetics of arsenate reduction would increase, as Cdc2 would be activated at a lower level. As observed in the results, the increase does not occur, therefore, Cdc25 could exert an activating role in this reduction independently of Cdc2 activation. On the other hand and despite these results, still remain to be cleared whether Cdc2 activity is regulated by Cdc25 in the reduction of arsenate, as has been described in the cell cycle [22].

These data show the complex mechanism by which *S. pombe* is able to reduce arsenate into arsenite. In the model we propose, Cdc25 and Cdc2 proteins play an activator and inhibitor role in the regulatory mechanism of the arsenate reduction, respectively. More studies are required in order to the molecular mechanism regulating arsenate into arsenite reduction, a key step for cell survival against this type of stress.

It is also interesting to notice that, although As (III) accumulates inside the cells, very little As (III) appears to accumulate in the growth media. This lack of arsenite accumulation could be explained by two different models:

- The export of As (III) to the cell exterior is not very efficient.
- There is an spontaneous oxidation of As (III) to As (V) in the growth media.

1. We consider that the second possibility is very unlikely because we have experienced very different responses in sensitivity from fission yeast to arsenate to arsenite. If arsenite would spontaneously oxidized to arsenate, the response to both forms of arsenic would be identical.

1. Therefore, we favour a model where the mechanisms of As (III) removal from the cytoplasm in fission yeast are not based in extracellular elimination, but on vacuolar accumulation, like the mechanism described previously [23], [24].
  We have described the mechanisms that lead to activation of the MAPK Spc1 by arsenate and the presence of an arsenate reductase activity in *S. pombe*. Future research will determine the regulation of this arsenate reductase activity and the possible interplay with other cellular stress response mechanisms.
